# The changes in the maize root cell walls after exogenous application of auxin in the presence of cadmium

**DOI:** 10.1007/s11356-023-28029-3

**Published:** 2023-07-07

**Authors:** Kristína Šípošová, Eva Labancová, Diana Hačkuličová, Karin Kollárová, Zuzana Vivodová

**Affiliations:** Institute of Chemistry, Slovak Academy of Sciences, Dúbravská cesta 9, 845 38 Bratislava, Slovakia

**Keywords:** Root cell walls, Maize, Auxin, Indole-3-butyric acid, Cadmium

## Abstract

Cadmium (Cd) is a transition metal and hazardous pollutant that has many toxic effects on plants. This heavy metal poses a health risk for both humans and animals. The cell wall is the first structure of a plant cell that is in contact with Cd; therefore, it can change its composition and/or ratio of wall components accordingly. This paper investigates the changes in the anatomy and cell wall architecture of maize (*Zea mays* L.) roots grown for 10 days in the presence of auxin indole-3-butyric acid (IBA) and Cd. The application of IBA in the concentration 10^−9^ M delayed the development of apoplastic barriers, decreased the content of lignin in the cell wall, increased the content of Ca^2+^ and phenols, and influenced the composition of monosaccharides in polysaccharide fractions when compared to the Cd treatment. Application of IBA improved the Cd^2+^ fixation to the cell wall and increased the endogenous concentration of auxin depleted by Cd treatment. The proposed scheme from obtained results may explain the possible mechanisms of the exogenously applied IBA and its effects on the changes in the binding of Cd^2+^ within the cell wall, and on the stimulation of growth that resulted in the amelioration of Cd stress.

**Supplementary Information:**

The online version contains supplementary material available at 10.1007/s11356-023-28029-3.

## Introduction

The cell wall is an essential feature of plants’ life. It is the layer that separates protoplast and the environment surrounding plants and considering its unique connection with the environment, it rapidly responds to environmental changes and participates both in signalling and defence reactions. During the contamination of the environment with heavy metals, cell walls can bind toxic cations and accumulate them into their structures (Krzesłowska 2011), which is a form of effective defence strategy.

One of the most studied heavy metals is cadmium (Cd), mostly because of its harmful effects on plants and also because of its negative impact on people’s health through the food chain (Huang et al. 2021). In plant cells, Cd induces severe oxidative stress, which leads to cell membrane damage, destruction of lipids and proteins, and may even cause cell death (Haider et al. 2021). Cd can also interfere with mineral nutrition status and causes disturbances in the uptake and distribution of mineral nutrients, which negatively affects plant biomass production (Huang et al. 2020). The cell wall is the first part of a plant that restricts Cd entry into the cytoplasm. This can be achieved through Cd^2+^ fixation within the cell wall polysaccharides or by physical barrier formation. Two different types (I and II) of cell walls are found in vascular plants. All dicotyledons and monocotyledons, except grasses, have type I that consists of a cellulose-xyloglucan network with the equivalent ratio of both units. Type I also contains a high amount of pectin and glycoproteins, such as extensins and glycine-rich proteins. However, the cell walls of grasses, including maize, are characteristic for type II which contains less pectin but has a higher amount of phenol than type I and contains different hemicelluloses, such as glucuronoarabinoxylan and mixed-linkage β-D-glucans (Carpita and Gibeaut 1993).

Auxins are plant hormones responsible for optimal plant growth, development, and defence mechanisms (Djami-Tchatchou et al. 2020). In our previous study, we found that exogenously applied indole-3-butyric acid (IBA) on maize plants affected the composition of the root cell wall, mainly the content of lignin and cellulose in the lignocellulose complex (Šípošová et al. 2019). However, these effects greatly depended on the concentration used. The application of auxins on plants is also considered as an interesting mitigation strategy to reduce Cd toxicity to plants (Rolón-Cárdenas et al. 2022). For example, exogenously applied IBA alleviated the Cd toxicity in maize roots, leading to improved plant oxidative status and mineral uptake (Šípošová et al. 2021). In our experiments, we used auxin IBA rather than indole-3-acetic acid (IAA), due to the higher stability of IBA in the solution (Nordström et al. 1991). It is also widely accepted, that IBA is an auxin precursor and can be easily converted to IAA in the process of IBA β-oxidation (Frick and Strader 2018).

According to numerous studies (Bashri and Prasad 2016; Šípošová et al. 2019; Rolón-Cárdenas et al. 2022), the application of auxins improves plant growth in stress conditions; however, there is a research gap that would clarify the involvement of these phytohormones in stress tolerance development. In the present study, we tried to verify two hypotheses pertaining to the following questions: (1) is auxin involved in the changes in the cell wall composition? (2) can the exogenous application of auxin increase the Cd-binding capacity of the cell wall of plants exposed to the Cd stress?

Although the application of auxins is recently gaining importance in studies that deal with different types of stresses on plants, most of the available studies about Cd and auxin interaction have been conducted on dicotyledonous plants (Zhu et al. 2013; Bruno et al. 2017), while there are not many studies about monocotyledonous plants including grasses. For the first time, the changes in the composition of root cell walls of maize (*Zea mays* L.) cultivated in the presence of Cd and IBA were studied. Maize plants have cell wall typical for grasses; thus, the present study of auxin and Cd effects on the cell walls provide new data that clarify the auxin involvement during plant response to abiotic stress.

## Material and methods

### Plant material and cultivation

Maize grains (*Zea mays* L., hybrid Almansa, obtained from RWA Slovakia s. r. o. Bratislava, Slovakia) were sterilised in 10% solution of detergent JAR (10 min), rinsed in tap water (15 min), immersed in 1.3 M sodium hypochlorite (10 min), and then rinsed again in tap water (15 min). The grains were imbibed in distilled water (3 h), followed by a germination on the wet perlite (3 days) in dark, at 25 °C and 70% ambient humidity. After the 3-day-long germination, the selected seedlings were transferred to containers for 10-day long hydroponic cultivation. The plants were grown in a controlled growth chamber (photosynthetic photon flux of 130–140 mmol m^−2^ s^−1^, temperature 25/20 °C, 70% humidity, and 16-h photoperiod). Full-strength Hoagland solution (Hoagland and Arnon 1950) at pH 6.2 was selected as the source of nutrients. The treatments were as follows: control, Cd in concentration 50 μM and in the form of Cd(NO_3_)_2_.4H_2_O (Cd treatment), IBA in concentration 10^−9^ M (IBA treatment), and a combination of Cd and IBA in concentrations: 50 μM Cd and 10^−9^ M IBA (Cd+IBA treatment). The selection of IBA concentration in this experiment was based on our previous results, where we studied the effects of exogenous IBA application on plant growth in the presence of Cd (Šípošová et al. 2021).

### The determination of the endogenous IAA concentration in roots

The concentration of endogenous IAA in roots was determined on the last day of cultivation according to the method of Demecsová et al. (2020). The 0.5-cm-long apical segments of 3 selected roots were homogenized in a cool mortar and extracted in 100% methanol, which contained 1 mM butylated hydroxytoluene. Then, the samples were left to incubate (4 h, 4 °C). After incubation, the samples were centrifuged (10 min, 13,700 g), and C18 columns were used for the purification of the extract. After that, the extract was methylated with trimethylsilyldiazomethane in hexane (ratio 7:3, 42 °C, 30 min). After the evaporation of the organic residues from the mixture, the residue was diluted with the solution of Tris-buffered saline. The quantification of IAA was determined by a competitive enzyme-linked immunosorbent assay (ELISA) (IAA immunoassay kit Olchemim, Czech Republic).

### Primary root thickness

The diameter of the primary root was observed at 30, 60 and 90% distances from the root apex (Šípošová et al. 2019). An inverted fluorescence DMI3000 B microscope (Leica Microsystems CMS GmbH, Germany), with excitation filter BP 450–490 nm, dichromatic mirror 510 nm, emission filter LP 515 nm, and ImageJ software (an open-source software designed for scientific image analysis) were used for all microscopic analyses.

### The visualisation of xylem and the development of the apoplastic barriers

The development of xylem and apoplastic barriers was determined by staining the cross sections of the primary root. The protoxylem, early and late metaxylem were visualised with phloroglucinol, while the Casparian bands were visualised with 0.2% berberine hemisulphate, and the suberin lamellae with 0.2% Fluorol Yellow 088 (Vaculík et al. 2012). The distances from the root apex to the first appearance of the xylem or the apoplastic barriers are expressed as a percentage of the root length.

### Isolation and purification of the root cell walls

The root cell walls were isolated according to modified methods of Šípošová et al. (2019). The dry plant material (10 g) was hydrated in distilled water, then homogenised by X-press (− 40 °C, 100 kPa), filtered through Miracloth, and lyophilised (Christ, BETA 2-8 LSC plus, PRAGOLAB s. r. o., Slovakia). The crude cell walls were rid of long fatty acids in the Soxhlet apparatus using chloroform/methanol (ratio 2:1, 4 h). The cleaned cell wall material was left to dry at room temperature, suspended in 50 mM phosphate buffer containing 0.5% α-amylase, and stirred at ambient temperature (24 h). In this process, α-amylase was used as an enzyme for the hydrolysis of starch in the samples. For the deactivation of α-amylase, the suspension was heated to 100 °C for 10 min, and afterwards, the suspension was filtered through glass Frit S4, dialysed (MCWO 3500, diameter 16 mm), and lyophilised. The crude cell walls were characterised by the content of carbohydrates, Klason lignin, proteins, and the concentration of Ca^2+^ and Cd^2+^.

The polysaccharide fractions (PFs) were isolated from the de-starched root cell walls by a two-step extraction. The PF I was extracted with 4% citric acid (80 °C, 60 min), and the PF II with 4.2 M KOH (60 °C, 90 min) (Šípošová et al. 2019). The PF III fraction constituted of the residue from the PF II. All extracts were precipitated by ethanol, dialysed and lyophilised. The PF I and II were characterised by their yield; the composition of monosaccharides; the contents of uronic acids, proteins, and phenols; and the concentration of Cd^2+^. The PF III was characterised by the yield, the content of the non-hydrolysable (cellulose) and hydrolysable part (non-cellulose polysaccharides), the content of Klason lignin, the content of the proteins, the monosaccharide composition of hydrolysable part, and the concentration of Cd^2+^.

### The analytical methods

The content of carbohydrates was determined by the modified phenol-sulphuric acid method of Dubois et al. (1956). Samples (100 μl) were thoroughly mixed with distilled water (100 μl), phenol (400 μl), and H_2_SO_4_ (2 ml) in a vortex mixer and left in the room temperature for 1 h. The absorption was measured spectrophotometrically at the wavelength 490 nm (Libra S6, Biochrom, UK). The glucose was used as a standard.

The protein content in the insoluble crude cell walls and PF III was calculated from the content of nitrogen as N (%) × 6.25. The protein content in the soluble fractions: PF I and PF II, was determined according to Bradford (1976) with bovine serum albumin used as a standard, and the absorption was measured spectrophotometrically at the wavelength 595 nm.

The content of Klason lignin (%) was determined according to a method of Schwanninger and Hinterstoisser (2002). The lyophilised samples (80 mg) with the addition of 72% H_2_SO_4_ (1.5 ml) and distilled water (36 ml) were hydrolysed under reflux (4 h). After hydrolysation, the samples were filtered through a glass Frit S4, washed with distilled water, and dried at 105 °C. The amount of lignin in the samples was determined gravimetrically as a percentage of the samples.

The concentrations of Ca^2+^ and Cd^2+^ were determined by atomic absorption microscopy (Perkin Elmer, model 1100) in the Geological Laboratories at the Faculty of Natural Sciences, Comenius University in Bratislava, Slovakia. The concentration of N was determined by elementary analysis using FLASH 2000 Organic Elemental Analyser (Thermo Fisher Scientific) at the Institute of Chemistry, Slovak Academy of Sciences, Slovakia.

The content of total phenols was determined according to Thaipong et al. (2006) with the Folin-Ciocalteu reagent (in ratio 1:1) and 20% Na_2_CO_3_. The gallic acid was used as a standard and the absorption was measured spectrophotometrically at the wavelength 750 nm.

The content of uronic acids was determined according to Blumenkrantz and Aboe-Hansen (1973). Sample (200 μl), containing from 3.88 to 33.0 μg galactouronic acid, was mixed with 0.125 M sodium tetraborate (3 ml) dissolved in concentrated H_2_SO_4_. First, the mixture was cooled in ice, then vigorously mixed in a vortex mixer, and then heated in a water bath (5 min, 100 °C). After the samples were cooled in an ice bath, the *m*-hydroxydifenyl reagent (50 μl) was added to the tubes. The absorbance of pink product was measured spectrophotometrically at the wavelength 520 nm. A sample in which the NaOH replaced the *m*-hydroxydifenyl (50 μl) reagent, was used as a reference.

The content of monosaccharides in PFs was determined by gas chromatography analysis (GC-MS). The samples (2 mg) were hydrolysed in trifluoroacetic acid (TFA) for 2 h; then, the TFA was removed by rotary evaporation using distilled water. After that, the samples were exposed to NaBH_4_ for 24 h in laboratory conditions and the excess of Na ions was eliminated by using dowex H^+^ ion exchange resins. The solution was vaporised and acetylated using pyridine and acetic anhydride for 24 h in laboratory temperature. The content of monosaccharides in the samples was obtained by GC-MS on a Trace GC Ultra (Thermo Scientific, USA) equipped with an SP-2330 fused silica capillary column (30 m × 0.25 mm × 0.2 μm) (Sulpeco, USA), a temperature program of 80 °C (4 min)–(8 °C/min)–160 °C (4 min)–(4 °C/min)–250 °C (12 min), and a flow rate of helium of 0.4 ml/min. The gas chromatograph was coupled with mass spectrometer TSQ Quantum XLS (Thermo Scientific, USA) with EI ionization under standard 70 eV, emission current 25 μA, ion source temperature 200 °C, full scan, mass range 40–450 m/z, and positive polarity (Englyst and Cummings 1984). The GC-MS analyses were conducted at the Institute of Chemistry, Slovak Academy of Sciences, Slovakia.

The content of cellulose in the PF III was calculated from the residue of the hydrolysable part of the lignocellulosic complex after the hydrolysis with 2 M trifluoroacetic acid under reflux for 2 h.

### The statistical analyses

Every experimental group and parameter were characterised by mean and standard error (SE). Microscopic experiments were repeated 10 times and chemical analyses 3 times. The differences between the experimental groups were evaluated by the Tukey test single-factor analysis of variance (ANOVA) in the statistical program Statistica, version 9.1, series 1009 (Statsoft, USA).

## Results

The effects of IBA and Cd on root growth, including primary root length, root anatomy, development of apoplastic barriers, differentiation of xylem, and endogenous concentration of IAA

The exposure of maize roots to the Cd in the hydroponic solution caused a significant inhibition of the primary root growth in comparison to other treatments (Table 1, Supplementary 1). The Cd treatment inhibited root length by 51.9%, when compared to the control plants, while the exogenous addition of IBA in the concentration 10^−9^ M (Cd+IBA treatment) stimulated root growth by 37.1% when compared to Cd treatment (Table 1). There was no significant difference in the root length between the control and the 10^−9^ M IBA treatment.

**Table 1:** The effects of Cd and IBA on the maize (*Zea mays* L.) primary root length. Different letters denote statistically significant differences in the parameters between the treatments at *p* < 0.05 according to the Tukey test

	Root length [cm]
Control	35.3 ± 0.7a
IBA	35.2 ± 1.3a
Cd	17.0 ± 0.5c
Cd + IBA	23.3 ± 1.1b

Different lowercase letters (a–c) denote statistically significant differences in the parameters between the treatments at *p* < 0.05 according to the Tukey

The application of IBA and Cd also influenced the root anatomy, such as the root diameter, the diameter of the stele, and the thickness of the root cortex (Table 2). Although no significant differences in the root diameter and thickness of the cortex near the root apex (30% distance from the root tip) were observed between all treatments, Cd, IBA, and Cd+IBA treatments influenced the diameter of the stele. The IBA, Cd, and Cd+IBA treatments increased this parameter by 21.5%, 44.9%, and by 26.4%, respectively, when compared to the control plants. At the 60% distance from the root tip, the Cd treatment significantly increased the root diameter as well as the diameter of the stele (by 11.9% and 44.4%, when compared to the control). However, the different impact of the treatments on the root anatomy was observed near the base (at the 90% distance from the root tip). The IBA treatment significantly increased the root diameter (by 16.3%) and heightened the thickness of the cortex (by 15.7%), when compared to the control. Contrary to the results from other parts of the root, near the root base, the Cd treatment decreased both the stele diameter (by 10.8%) and the thickness of the cortex (by 18.8%) in comparison to the control plants.

**Table 2:** The effects of Cd and IBA on the root and stele diameter, and the cortex thickness of maize (*Zea mays* L.). The cross section of primary roots was made at the distances of 30%, 60%, and 90% from the root apex. Different letters denote statistically significant differences in the parameters between the treatments at *p* < 0.05 according to the Tukey test

	Root diameter [μm]		
	30%	60%	90%
Control	524.5 ± 22.9a	654.3 ± 15.6b	943.0 ± 5.9b
IBA	511.9 ± 30.8a	632.5 ± 35.4b	1096.8 ± 10.2a
Cd	549.8 ± 22.1a	732.2 ± 0.6a	930.8 ± 47.0b
Cd + IBA	526.8 ± 10.5a	619.8 ± 22.0b	924.3 ± 30.6b
	Stele diameter [μm]		
	30%	60%	90%
Control	180.5 ± 11.8c	239.0 ± 5.1b	448.0 ± 5.3a
IBA	219.3 ± 10.8b	262.3 ± 28.1b	424.6 ± 17.5ab
Cd	261.6 ± 6.3a	345.1 ± 3.3a	399.6 ± 23.1b
Cd + IBA	228.2 ± 6.5b	255.9 ± 10.1b	459.2 ± 15.9a
	Thickness of cortex [μm]		
	30%	60%	90%
Control	145.3 ± 6.6a	202.7 ± 6.6a	252.0 ± 8.0b
IBA	147.2 ± 6.3a	200.1 ± 21.7a	291.6 ± 4.2a
Cd	157.4 ± 12.1a	182.6 ± 1.3a	204.5 ± 11.4c
Cd + IBA	151.5 ± 5.1a	178.2 ± 8.2a	229.5 ± 11.5bc

Different lowercase letters (a–c) denote statistically significant differences in the parameters between the treatments at *p* < 0.05 according to the Tukey

**Fig. 1:**
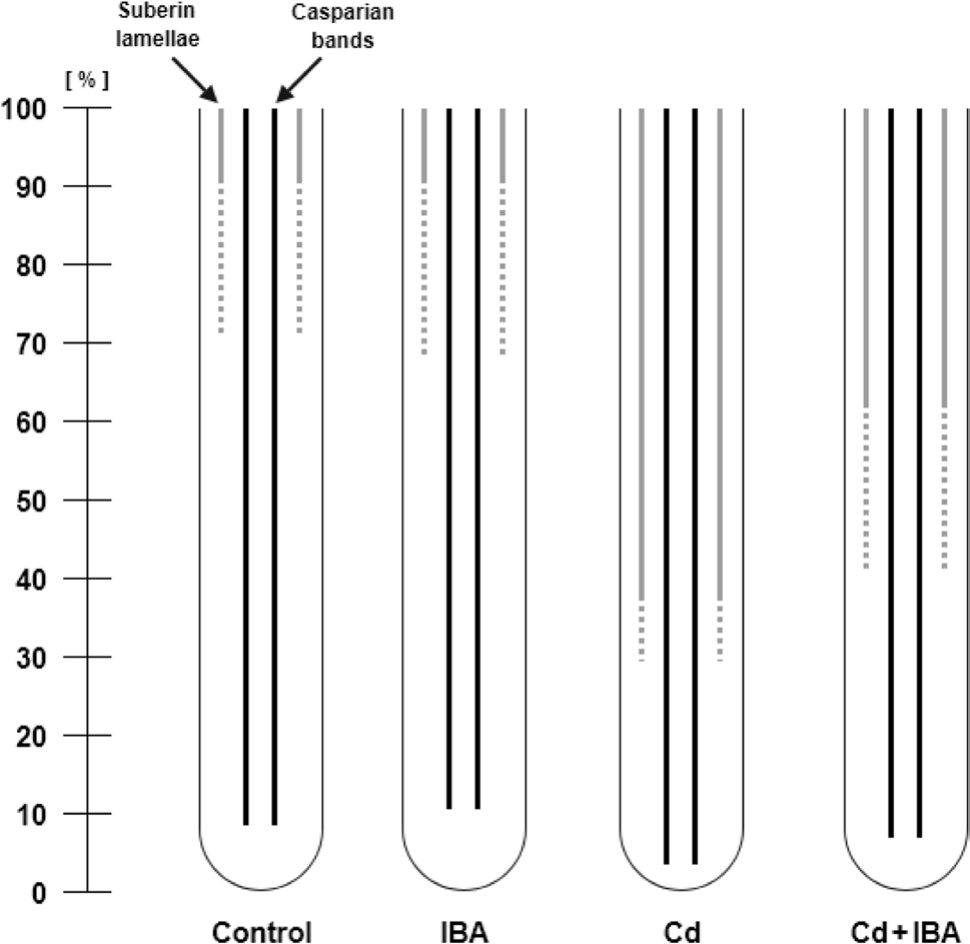
The scheme of the development of the apoplastic barriers (Casparian bands, suberin lamellae) in the endodermis of the primary roots of maize (*Zea mays* L.). Solid black lines represent the full development of Casparian bands, solid grey lines represent the fully developed suberin lamellae and the broken grey lines represent the partially developed suberin lamellae. The distance from the root apex is expressed as a percentage of the total root length, due to the different root lengths in control plants and other treatments. Different letters denote statistically significant differences in the parameters between the treatments at *p* < 0.05 according to the Tukey test

The development of apoplastic barriers (Casparian bands, suberin lamellae) in the root endodermis was evaluated. The Cd treatment caused earlier development of Casparian bands (CBs) in comparison to the control plants (by 5.9%) (Fig. 1, Supplementary 2). However, the CBs in the Cd+IBA treatment were developed later when compared to the Cd treatment (by 3.3%), and no significant differences were detected between the control plants and the 10^−9^ M IBA treatment. The Cd treatment caused an earlier development of suberin lamellae (SL) when compared to the control plants (by almost 50%) (Fig. 1, Supplementary 2). The roots of Cd+IBA treatment developed SL later than the roots of the Cd treatment (partially developed SL by 11.3%, fully developed SL by 23.9%) and the 10^−9^ M IBA treatment did not significantly affect SL development when compared to the control plants.

**Fig. 2:**
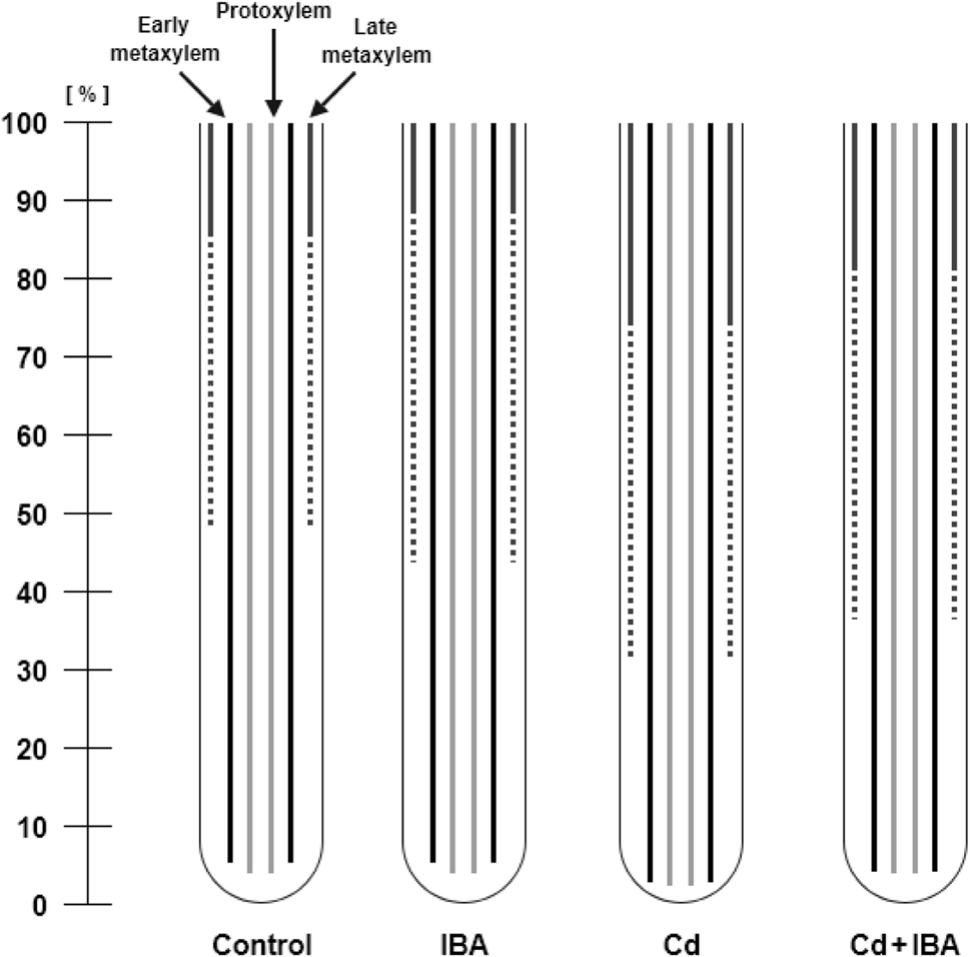
The scheme of the lignification of the protoxylem, early and late metaxylem in the primary roots of maize (*Zea mays* L.). Solid grey lines represent the full development of protoxylem, solid black lines represent the fully developed early metaxylem, the broken dark grey lines represent the partially late metaxylem, and the solid dark grey lines represent the fully developed late metaxylem. The distance from the root apex is expressed as a percentage of the total root length, due to the different root lengths in control plants and other treatments. Different letters denote statistically significant differences in the parameters between the treatments at *p* < 0.05 according to the Tukey test

The effects of IBA and Cd on the differentiation of protoxylem (PX), early (EM) and late metaxylem (LM) were also determined (Fig. 2, Supplementary 3). The Cd treatment caused an earlier differentiation of PX (by 1.5%) and EM (by 2.2%) when compared to the control. On the other hand, the Cd+IBA treatment delayed the differentiation of both PX and EM when compared to the Cd treatment (by 1.5% and 1.5%, respectively). The LM partially developed earlier in both Cd and Cd+IBA treatments than in control plants (by 16.8% and 11.9%, respectively). On the other hand, the fully developed LM was detected significantly closer to the apex only in the Cd treatment when compared to the control (by 11.4%). The 10^−9^ M IBA treatment did not significantly influence the lignification of any type of determined xylem.

**Fig. 3:**
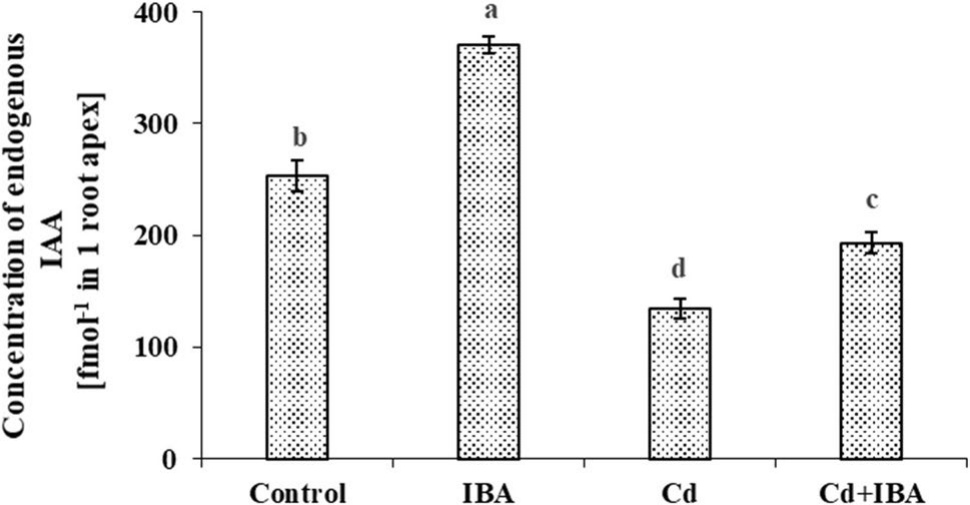
The concentration of endogenous IAA (fmol per root apex) in maize (*Zea mays* L.) roots. Different letters denote statistically significant differences in the parameters between the treatments at *p* < 0.05 according to the Tukey test

The exogenous application of sole IBA significantly elevated the endogenous IAA concentration when compared to the control plants (by 46.7%), while Cd treatment decreased this parameter (by 47.6%) (Fig. 3). However, we detected a significant increase of IAA concentration in the roots treated by Cd+IBA (by 43.0%) when compared to the plants of Cd treatment.

### The effects of IBA and Cd on the root cell wall and polysaccharide fractions

The primary cell wall of plants is mainly composed of polysaccharides, functional proteins, and a small amount of phenols. In our experiment, no significant differences in the content of the total carbohydrates in the de-starched crude cell walls between the control plants and other treatments were observed; however, the changes were detected in other parameters (Table 3). The content of proteins was significantly decreased by the Cd treatment (by 8.4%), and it was positively influenced by the 10^−9^ M IBA treatment (elevated by 22.7%) when compared to the control plants. Only the Cd treatment caused a significant change in the content of Klason lignin (increased by 33.3% in comparison to the control plants). The only significant difference in the concentration of Ca^2+^ was detected between the control plants and the Cd treatment (decreased by 33.1%). The concentration of Cd^2+^ in the Cd+IBA treatment was significantly higher (by 11.5%) when compared to the Cd treatment.

**Table 3:** The characterisation of the maize (*Zea mays* L.) root cell walls. All values were calculated per dry mass. Different letters denote statistically significant differences in the parameters between the treatments at *p* < 0.05 according to the Tukey test

	TC [%]	Proteins [%]	Klason lignin [%]	Ca^2+^ [μg/g]	Cd^2+^ [μg/g]
Control	78.2 ± 1.6a	11.9 ± 0.1b	10.2 ± 1.3b	2253.7 ± 2.3a	n.d.
IBA	77.5 ± 2.5a	14.6 ± 0.2a	11.9 ± 0.3ab	2241.5 ± 18.3a	n.d.
Cd	77.7 ± 3.6a	10.9 ± 0.2c	13.6 ± 0.2a	1507.7 ± 3.3b	2792.0 ± 21.8b
Cd + IBA	80.0 ± 2.9a	11.3 ± 0.2bc	11.0 ± 0.2b	2249.3 ± 2.3b	3112.5 ± 34.2a

*n.d.* not detected, *TC* total content of carbohydrates

Different lowercase letters (a–c) denote statistically significant differences in the parameters between the treatments at *p* < 0.05 according to the Tukey

#### The polysaccharide fraction I

The polysaccharide fractions were extracted from the de-starched crude cell walls by a two-step procedure (see the methods). Cd treatment greatly affected all PF I parameters (Table 4) which included an increased yield (by 9.4%). We detected an increase in rhamnose (Rha), fucose (Fuc), and galactose (Gal) contents (increased by 85.4%, 83.5%, and 56.6%, respectively), when compared to the control. Further, we detected decreased contents of arabinose (Ara) and xylose (Xyl) (decreased by 37.8% and 40.0%, respectively), and no significant differences in the content of glucose (Glc) and mannose (Man). The content of uronic acids (UA) and phenols was two times as high in the Cd treatment as in the control, while the content of proteins increased by 35.6%.

**Table 4:** The characterisation of the polysaccharide fraction I (PF I) in maize (*Zea mays* L.) roots by the yield, the content of monosaccharides, uronic acids, proteins, phenols, and the concentration of Cd^2+^. Different letters denote statistically significant differences in the parameters between the treatments at *p* < 0.05 according to the Tukey test

	Yield [%]	Monosaccharides [%]								Proteins [%]	Phenols [%]	Cd^2+^ [μg/g]
		Xyl	UA	Ara	Gal	Rha	Fuc	Glc	Man			
		(Glucurono)arabinoxylan						Glucomannan				
				Pectin								
				Arabinogalactan I, II								
Control	3.2	30.5 ± 0.9a	1.6 ± 0.0c	28.8 ± 0.4b	15.2 ± 0.8c	4.8 ± 0.2b	9.1 ± 0.5b	11.0 ± 1.0a	3.0 ± 0.4b	5.9 ± 0.1c	0.2 ± 0.1c	n.d.
IBA	3.2	25.0 ± 1.0b	2.3 ± 0.1b	19.8 ± 0.9c	20.6 ± 0.7b	5.1 ± 0.5b	9.9 ± 0.5b	11.4 ± 1.1a	5.9 ± 0.8ab	4.5 ± 0.2d	0.1 ± 0.1c	n.d.
Cd	3.5	18.3 ± 0.6c	3.2 ± 0.1a	17.9 ± 0.4c	23.8 ± 0.5a	8.9 ± 0.5a	16.7 ± 0.6a	11.5 ± 0.6a	3.4 ± 0.2b	8.0 ± 0.4b	0.4 ± 0.1b	606.0 ± 1.0a
Cd + IBA	4.3	23.6 ± 0.4b	2.9 ± 0.1a	32.6 ± 0.7a	19.9 ± 0.4b	4.3 ± 0.3b	4.3 ± 0.4c	11.7 ± 0.8a	4.2 ± 0.4ab	9.7 ± 0.2a	0.7 ± 0.1a	512.3 ± 0.7b

*n.d.* not detected

Different lowercase letters (a–c) denote statistically significant differences in the parameters between the treatments at *p* < 0.05 according to the Tukey

The Cd+IBA treatment increased the yield of PF I by 23.0% when compared to the Cd treatment, and significantly changed the contents of all monosaccharides, except Man and Glc (Table 2). The Cd+IBA treatment increased the content of Ara and Xyl (by 82.1% and 29.0%, respectively) and decreased the content of Rha, Fuc, and Gal (by 51.7%, 74.3%, and 16.4%, respectively) when compared to the Cd treatment. In addition, an increase in the content of proteins and phenols was detected (increased by 21.3% and 75.0%, respectively).

#### The polysaccharide fraction II

The second polysaccharide fraction (PF II) was composed mainly of hemicellulose polysaccharides and a small amount of alkali-soluble pectin. The Cd treatment increased the yield of PF II and affected the monosaccharide composition (Table 5). The Cd treatment increased the content of Glc and Xyl (by 152.3% and 41.0%, respectively), and decreased the content of Rha, Fuc, and Ara (by 58.4%, 75.0%, and 55.9%, respectively) when compared to the control plants. The presence of Cd in the medium also increased the content of UA (by 40.5%), proteins (by 29.6%), and phenols (by 50.0%) (Table 5).

**Table 5:** The characterisation of the polysaccharide fraction II (PF II) in maize (*Zea mays* L.) roots by the yield, the content of monosaccharides, uronic acids, proteins, phenols, and the concentration of Cd^2+^. Different letters denote statistically significant differences in the parameters between the treatments at *p* < 0.05 according to the Tukey test

	Yield [%]	Monosaccharides [%]								Proteins [%]	Phenols [%]	Cd^2+^ [μg/g]
		Glc	Xyl	UA	Ara	Gal	Rha	Fuc	Man			
			(Glucurono)arabinoxylan									
		(1→3,1→4)-Glucan		Pectin								
					Arabinogalactan I, II							
Control	24.4	4.4 ± 0.2c	43.9 ± 0.6b	8.4 ± 0.6c	41.3 ± 0.5a	7.8 ± 0.3a	1.2 ± 0.1b	0.8 ± 0.1a	n.d.	5.4 ± 0.2c	0.8 ± 0.1c	n.d.
IBA	29.6	4.3 ± 0.1c	43.5 ± 0.3b	10.0 ± 0.1c	41.8 ± 0.7a	7.3 ± 0.2a	2.4 ± 0.1a	0.9 ± 0.1a	n.d.	5.7 ± 0.6c	0.9 ± 0.1c	n.d.
Cd	31.7	11.1 ± 0.5a	61.9 ± 0.8a	11.8 ± 0.4b	18.2 ± 0.2c	7.6 ± 0.3a	0.5 ± 0.1c	0.2 ± 0.1b	n.d.	7.0 ± 0.2b	1.2 ± 0.1b	2594.3 ± 2.0b
Cd + IBA	32.6	6.2 ± 0.2b	61.7 ± 0.7a	14.5 ± 0.7a	23.9 ± 0.6b	7.4 ± 0.2a	0.6 ± 0.1c	0.3 ± 0.1b	n.d.	8.2 ± 0.2a	1.4 ± 0.1a	3862.7 ± 4.8a

*n.d.* not detected

Different lowercase letters (a–c) denote statistically significant differences in the parameters between the treatments at *p* < 0.05 according to the Tukey

On the other hand, the Cd+IBA treatment increased the content of Ara (by 31.3%) and decreased the content of Glc (by 44.1%) when compared to the Cd treatment. Further, the content of UA, proteins, and phenols increased (by 22.9%, 17.1%, and 16.7%, respectively).

#### The polysaccharide fraction III

The third polysaccharide fraction (PF III) contained the residue of the cell walls—mainly the lignocellulosic complex and a small amount of hemicellulose. In our study, non-hydrolysable part of this fraction represents cellulose and hydrolysable part represents hemicellulose. The Cd treatment significantly increased the content of Glc, Gal, and Ara (by 25.9%, 55.6%, and 58.7%, respectively) and decreased the content of Xyl (by 36.3%) when compared to the control plants (Table 6). Higher contents of lignin and hemicelluloses and lower content of cellulose were found in the PF III of the Cd treatment (increased by 37.3% and 56.8%, and decreased by 28.0%, respectively) when compared to the control. The Cd+IBA treatment increased the content of Glc and Gal (by 38.2% and 31.3%, respectively) and decreased the content of Ara (by 33.3%) when compared to the Cd treatment. Contrary to the Cd treatment, a higher content of cellulose and lower content of hemicellulose (increased by 13.0% and decreased by 14.2%, respectively) was detected in the Cd+IBA treatment.

**Table 6:** The characterisation of the polysaccharide fraction III (PF III) in maize (*Zea mays* L.) roots by the yield, the composition of monosaccharides, the content of Klason lignin, proteins and the concentration of Cd^2+^. Different letters denote statistically significant differences in the parameters between the treatments at *p* < 0.05 according to the Tukey test

	Yield [%]	NHP [%]	Klason lignin [%]	HP [%]	Monosaccharides [%]							Proteins [%]	Cd^2+^ [μg/g]
					Man	Glc	Fuc	Rha	Gal	Ara	Xyl		
						Glucan			Glucuronoarabinoxylan				
Control	49.0	62.1 ± 0.5a	21.7 ± 1.4c	16.2 ± 1.0c	n.d.	13.5 ± 0.5c	n.d.	n.d.	7.2 ± 0.7c	22.5 ± 0.8b	56.7 ± 1.7a	n.d.	n.d.
IBA	47.1	56.8 ± 0.6b	23.6 ± 1.8bc	19.6 ± 1.0bc	n.d.	15.1 ± 0.8bc	n.d.	n.d.	6.0 ± 0.6c	36.5 ± 0.7a	42.4 ± 1.3b	n.d.	n.d.
Cd	43.8	44.7 ± 1.3d	29.8 ± 0.6a	25.4 ± 0.8a	n.d.	17.0 ± 0.5b	n.d.	n.d.	11.2 ± 0.2b	35.7 ± 0.4a	36.1 ± 1.0c	n.d.	280.8 ± 0.3a
Cd + IBA	51.4	50.5 ± 0.7c	27.7 ± 0.9ab	21.8 ± 0.5b	n.d.	23.5 ± 0.5a	n.d.	n.d.	14.7 ± 0.4a	23.8 ± 0.8b	38.1 ± 1.0c	n.d.	193.0 ± 0.1b

*n.d.* not detected, *HP* hydrolysable part of PF III, *NHP* non-hydrolysable part of PF III

Different lowercase letters (a–c) denote statistically significant differences in the parameters between the treatments at *p* < 0.05 according to the Tukey

### The effects of IBA and Cd on the concentration of Cd in the polysaccharide fractions

**Fig. 4:**
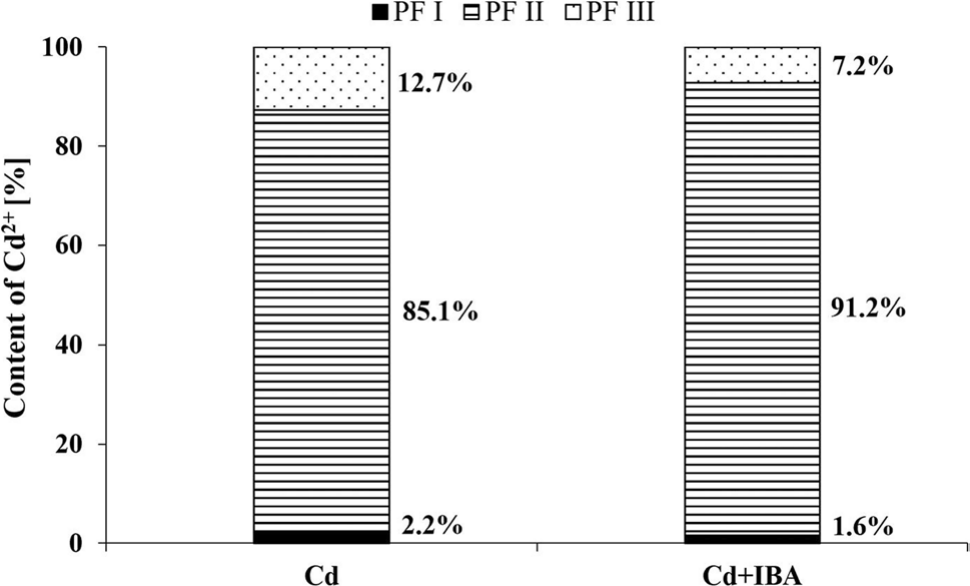
The distribution of Cd^2+^ in the polysaccharide fractions (PF I–PF III) extracted from the root cell walls of maize (*Zea mays* L.)

Cd was not distributed equally in different fractions (Fig. 4). In the Cd treatment, the highest amount of Cd (85.1%) was found in the PF II followed by PF III (12.7%) and PFI (2.2%). The Cd+IBA treatment changed the distribution of Cd, when compared to Cd treatment. It increased the content of Cd in the PF II (by 6.1%) and decreased its content in the PF III and PF I (by 5.5% and 0.6%, respectively).

## Discussion

### The effects of IBA and Cd on root growth, including primary root length, root anatomy, development of apoplastic barriers, and differentiation of xylem

In our present study, we ascertained that Cd in 50 μM concentration negatively affected plant growth and primary root elongation (Tables 1 and 2, Supplementary 1), which is also in accordance with other reports (Dresler et al. 2015; Šípošová et al. 2021; Matayoshi et al. 2022). Root growth is defined by high metabolic activity; therefore, roots are very sensitive to the stress induced by heavy metal contamination in the environment (Finger-Texeira et al. 2010). One of the possible ways how could the presence of Cd in the substrate inhibit root growth is via the reduction of mitotic activity, induction of chromosome disorders, and nuclear abnormalities in cells of the apical meristem (Wang et al. 2014). Cd can also bind to proteins, which leads to the disruption of their structure and inhibition of their activity (Haider et al. 2021). According to other studies (Bliou et al. 2005; Bruno et al. 2017), the inhibitory effect of Cd on the root growth might be also linked with the impairment of auxin accumulation at the root tip, which causes the reduction of the meristem size and activity.

Several studies have reported the positive influence of auxin application on plants exposed to Cd stress (Zhu et al. 2013; Chen et al. 2020; Zhang et al. 2020); however, the exact mechanism through which auxins increase the plant’s tolerance against Cd is still not properly understood (Rolón-Cárdenas et al. 2022). Cd reduces the concentration of endogenous IAA (Fig. 3) through the stimulation of IAA-oxidase activity, which induces IAA degradation (Bashri and Prasad 2016). Cd also affects the expression of auxin-related genes, including auxin biosynthesis (Yue et al. 2016; Zhan et al. 2017), its transport into the cell (Huang et al. 2023), and downstream auxin response (Yue et al. 2016). Here, the application of IBA on maize roots probably elevated the depleted level of IAA caused by Cd, thus improved not only the root growth but also the oxidative status of the plants and the uptake of nutrients (Šípošová et al. 2021).

According to Frick and Strader (2018), IBA found in plants at a lower level than IAA and is very difficult to detect. IBA acts as IAA precursor and can be converted to IAA; therefore, the concentration of endogenous IAA was evaluated. We ascertained that the IAA concentration increased in the IBA and the Cd+IBA treatments compared to the control and Cd treatment, respectively.

According to many studies, plants that are exposed to Cd have thicker but shorter roots (Lux et al. 2011; Ismael et al. 2019) due to the toxic effects of Cd on the mitosis in the meristem (Seth et al. 2008). The enlargement of cortical tissues and parenchymal cells might also enhance the resistance of tissues to the radial flow of water and solutes (Lux et al. 2011; Haider et al. 2021). In addition, the thickening of the cell walls that was induced by Cd stress can enhance the root Cd retaining capacity and limit Cd translocation to the shoot (Zhang et al. 2022). In the present study, the IBA (10^−9^ M) influenced the proportions of the roots, which might be connected to its positive influence on root growth in stress conditions (Table 2). Similar, IBA in various concentrations changed the proportions of the roots in non-stress conditions; however, its influence on root anatomy greatly depended on the particular concentration and its impact on root growth (Šípošová et al. 2019). Auxins are known for their stimulatory effects on cell division, vascular differentiation, and root initiation, as well as their ability to induce cell elongation (Normanly et al. 2010; Khadr et al. 2020). They also activate the expression of cell wall-related genes and stimulate the synthesis of proton pumps, which leads to apoplast acidification (Perrot-Rechenmann 2010) and activation of the cell wall-loosening proteins responsible for wall enlargement (Majda and Roberts 2018). The changes in the root anatomy might be connected to the improved level of IAA in roots and modified cell wall composition.

The earlier development of apoplastic barriers in the root apex is a defence mechanism of plants against stress because the presence of these barriers in the endodermis can hinder the movement of water and dissolved ions through the apoplastic pathway (Vaculík et al. 2012; Vatehová et al. 2016). Our results are in agreement with the studies of Vaculík et al. (2012) and Zhang et al. (2022), where apoplastic barriers in plants exposed to Cd also developed closer to the root tips (Fig. 1, Supplementary 2). In our previous work (Šípošová et al. 2019), the exogenous application of IBA did not affect the development of CBs, even though IBA influenced maize growth. However, the application of IBA in inhibitory concentration (10^-7^ M) stimulated the development of SL closer to the root apex, which suggests the presence of stress. In the present study, 10^−9^ M IBA treatment did not significantly modify the development of apoplastic barriers; however, its application on Cd-stressed maize changed this parameter (Fig. 1, Supplementary 2). The delay of barrier development in roots of the Cd+IBA treatment can be linked with improved root elongation, greater biomass, higher level of IAA, and the positive effect of IBA on the prevention of Cd transport through the extracellular space. The changes in the cell wall composition and formation of new sites that retain Cd could have diminished the necessity of earlier apoplastic barrier development in the Cd+IBA treatment.

It is well known that Cd is transported from roots to shoots by longitudinal translocation via the system of xylem vessels (Vaculík et al. 2012), and that the presence of Cd in plants accelerates not only root maturation but the development of xylem as well (Vitória et al. 2001). Ďurčeková et al. (2007) found that Cd induced the changes in the production of H_2_O_2_ and peroxidase activity in the region 2–8 mm from the root tip, which is associated with earlier maturation of xylem elements. Auxins can stimulate xylem tissue differentiation in non-stress conditions (Aloni et al. 2006); however, their effect depends on the concentration used (Šípošová et al. 2019). The development of vascular tissues is controlled by polar IAA movement in the vascular cambium along the plant body from hydathodes of young leaves downward to root tips (Aloni et al. 2006). Khadr et al. (2020) determined that IBA application in concentrations 100 and 150 μmol increased the number and area of xylem vessels, and decreased the content of lignin in carrot (*Daucus carota* L.) roots. Changes in the xylem vessel maturation might be attributed to the asymmetric distribution of specific transport proteins regulated by vesicle trafficking which are usually confined to the apical/basal end-poles of auxin-transporting cells (Normanly et al. 2010). According to Khan and Chaundhry (2005) and Chaudhry and Khan (2007), exogenously applied auxin stimulates mitosis of cambial cells leading to the differentiation of new xylem cells in *Lagenaria siceraria* and *Cucumis sativum* plants treated with the toxic element.

### The effects of IBA and Cd on the root cell wall and polysaccharide fractions

The cell wall is the first contact point of the cell with the environment and acts like a plastic barrier that suppresses Cd entry and transport to the cytoplasm (Krzesłowska 2011; Parrotta et al. 2015). In our previous study (Šípošová et al. 2021), we found that exogenously applied IBA on roots in the same concentration as used in this study (10^−9^ M) decreased the concentration of Cd from 7420 μg/g of dry weight to the 4970 μg/g. Hence, in the present study, we focused on the changes in the cell wall composition after IBA application on plants exposed to Cd, specifically on the Cd^2+^ fixation into the cell wall structure as a defence mechanism.

In our study focused on the crude cell wall composition, the content of carbohydrates did not change, the content of proteins and Ca^2+^ decreased, and the content of lignin increased under Cd stress (Table 3). Similar results were also observed in the study conducted by Vatehová et al. (2016). According to Vogel (2008), the cell wall proteins might be strongly or loosely attached to polysaccharides or present as soluble proteins. Structural proteins are less abundant in the grasses than in the dicots, and their function and involvement in the cell wall remodeling under abiotic stress are still unknown. The gaps in the knowledge might be explained by the difficulty of cell wall protein isolation without contamination by cytoplasmatic proteins or proteins from the other cell wall components (Ghahremani et al. 2016). The increased content of lignin in the Cd treatment might be related to the earlier development of apoplastic barriers and thickening of the cell wall (Liu et al. 2018). Furthermore, the deposition of lignin in the cell wall of the root endodermis may inhibit the transport of Cd into the xylem. Ca^2+^ is an essential macronutrient with a dual function in plants: it acts as a structural component of the cell wall and membranes, and as an intracellular secondary messenger in a variety of cellular processes (Thor 2019). In the cell walls, Ca^2+^ also participates in cross-linking negative charges of the carboxylic residues of pectin, which contributes to the cell wall rigidity (Krzesłowska 2011). The elevated concentration of Cd^2+^ in the cell wall after application of the Cd+IBA (when compared to the Cd treatment) might be explained by the increased binding of Cd^2+^ in the crude cell wall. The Ca^2+^ and the Cd^2+^ ions share the same binding sites in the cell wall and Cd^2+^ as the element with both higher electronegativity and molar mass might have replaced the Ca^2+^ in the cell wall structure (Krzesłowska 2011).

In the next part of our study, we conducted a detailed analysis of the cell wall of maize roots, and from the two-step procedure, we isolated three polysaccharide fractions of the crude cell walls. The first polysaccharide fraction (PF I) was extracted with 4% citric acid which was used to obtain pectin from the plant cell walls (Šípošová et al. 2019). The citric acid was selected as a more suitable reagent due to its higher extraction yield when compared to the extraction by hot water (Fang et al. 2020). The second polysaccharide fraction (PF II) was extracted with 4.2 M KOH, which was used to obtain the hemicellulose fraction (Xiao et al. 2001) and alkali-soluble pectin (Gawkowska et al. 2018). This type of pectin is stronger bound to other polysaccharides than the pectin extracted by hot water or citric acid. The third polysaccharide fraction (PF III) was a residual fraction obtained after the second extraction and it consisted of the insoluble lignocellulosic complex.

In our study, we determined that PF I is a mix of pectin polysaccharides (Rha, Fuc, Ara, Gal, and UA) and some water-soluble hemicelluloses (arabinoxylan (Ara), Xyl; glucomannan (Glc), Man; arabinogalactan (Ara), Gal) (Table 4). Xu et al. (2007) yielded a similar mixture of polysaccharides from ryegrass (*Lolium perenne* L.) after the extraction with hot water. The main component of pectin is galacturonic acid which can bind divalent cations (Krzesłowska 2011). The high contents of Fuc, Rha, and UA in the Cd treatment indicated a high content of pectin. However, the Cd+IBA treatment decreased the content of the abovementioned monosaccharides, when compared to the Cd treatment. Pectin accounts for 20–35% of the total polysaccharides content in dicots (O’Neill and York 2003) and the accumulation of pectin in their cell walls is a defence mechanism against various stressors, e.g., toxic metals (Krzesłowska 2011). Cd changes the methylesterification of pectins through the modification of pectin methylesterase (PME) activity (Szerement and Szatanik-Kloc 2022); however, its influence depends on the plant species and Cd concentration. Unlike the cell walls of dicots, the cell walls of grasses contain less than 5% of pectin (O’Neill and York 2003) and the reason behind this difference has not yet been fully explained. However, it appears that the cells of monocots have the same reaction against Cd toxicity as dicots (Yu et al. 2020) and accumulation of pectin has probably similar role. Exogenous application of auxin stimulates the activity of PME (Braybrook and Peaucelle 2013) which results in higher de-methylesterification of pectin; and thus, could alleviate the damage caused by Cd.

In the present study, the Cd+IBA treatment increased the content of Ara and Xyl, monosaccharides that create arabinoxylan, when compared to the Cd treatment. The main function of soluble arabinoxylan is to cross-link cell wall components (Bento-Silva et al. 2018) including phenols (Izydorczyk 2012). However, some evidence is available about its involvement during stress, e.g., heat and drought stress (Rakszegi et al. 2014). In our experiments, the content of phenols increased both in the Cd treatment and in the Cd+IBA treatment. Hence, the increased content of arabinoxylan in the cell wall could have contributed to the embedding of phenols in the cell wall. Phenols are involved in metal stress responses in plants because they contain hydroxyl and carboxyl groups that can bind toxic metals (Kısa et al. 2016).

The glucomannans are present only in small amounts in the type II cell walls (Carpita et al. 2001) while they are more dominant in dicots. Our results also suggest their minimal engagement in stress responses of grasses because the content of Glc and Man did not significantly change in the Cd- and Cd+IBA-treated plants.

The PF I contained some portion of proteins, probably arabinogalactan proteins, which have an important role in plant embryogenesis, plant development, and during defence against biotic stress. Arabinogalactan proteins can act as structural components and/or signalling molecules that initiate the defence mechanisms, such as the colonization of the root tip by beneficial microbes (Nguema-Ona et al. 2013).

We determined that the monosaccharides dominantly present in PF II were Xyl, Ara, and UA, which form the main polysaccharide that creates type II cell wall – glucuronoarabinoxylan (GAX) (Table 5) (Carpita et al. 2001). Xylose acts as the main chain of GAX and its content was not affected by the application of IBA on Cd-stressed plants (the Cd+IBA treatment) when compared to the Cd treatment. However, taking in account the ratio of two important GAX components—Ara:Xyl—the differences among the treatments are visible. The Cd treatment changed the Ara:Xyl ratio to 1:3.4 and the Cd+IBA treatment to 1:2.6 in contrast to the control plants (1:1). The changed ratio of Ara:Xyl in the Cd+IBA treatment suggested a higher branching and substitution of GAX main chain when compared to the Cd treatment (Carpita 1996; Carpita et al. 2001). The highly substituted GAX cross-links with the non-cellulosic polysaccharides of the cell wall and is associated with improved growth of plants, while the low substituted GAX cross-links with the cellulose microfibrils and is associated with the increased cell wall rigidity. This is in accordance with our results of maize growth as the Cd+IBA treated plants showed improved growth when compared to the Cd treatment. The increased rigidity of the maize cell walls is a defence mechanism against the toxic metal stress (Degenhardt and Gimmler 2000). The exogenous application of auxin alleviated the toxic effect of Cd by the improvement of nutrient uptake and activation of antioxidant mechanisms (Šípošová et al. 2021) which possibly changed the cell wall response to the Cd presence. Hence, the necessity to increase the cell wall rigidity by lower GAX substitution in the Cd+IBA treatment was not as acute as in the Cd treatment. Similar to PF I, a high content of phenols in PF II and their linkage with GAX (Kısa et al. 2016) actively participated in the defence against the Cd stress.

Mixed-linkage glucans are typical polysaccharides of the cell wall type II extractable with alkali reagents (Carpita 1984). In the present study, the Cd treatment increased the content of Glc, which indicates a higher content of glucans, while the Cd+IBA treatment had opposite effect. Glucans connect to cellulose microfibrils during plant growth (Buckeridge et al. 2004). Hoson et al. (1992) found that the glucans underwent degradation during IAA-induced growth and implied the importance of the glucan degradation. Furthermore, Kim et al. (2018) reported that the increased content of glucans in the cell wall can also cause irregularities during plant growth and development.

We determined that PF II contained a small amount of alkali-soluble pectin that connects other cell wall polysaccharides via a covalent ester bond (Gawkowska et al. 2018). Both the Cd and the Cd+IBA treatments decreased the content of monosaccharides that occur only in pectin (such as Rha and Fuc) when compared to the control plants. Moreover, the pectin content in the cell wall of grasses is very low (less than 5%) (O’Neill and York 2003), and alkali-soluble pectin, which has a dominant role in cell wall surface charging (Szatanik-Kloc et al. 2017), equals only to 1%.

In our study, PF III was composed of non-hydrolysable and hydrolysable parts as well as Klason lignin (Table 6). Our results confirm that plants growing under stress conditions contain less cellulose and more lignin than the plants growing in non-stress conditions. Similar effect was confirmed in the grasses that were exposed to salinity (Tiwari et al. 2018; Oliveira et al. 2020), drought (Tiwari et al. 2018), and toxic metals (Vatehová et al. 2016). The deficit of some mineral nutrients such as S, N, and P can decrease the cellulose content in the cell walls (Parrotta et al. 2015). Although no evidence is currently available about the direct effect of Cd on cellulose synthesis, the application of Cd can decrease the uptake of essential nutrients, including S, N, and P (Šípošová et al. 2021). In the present study, the Cd and the Cd+IBA treatment influenced the ratio of lignin and cellulose, which might be connected to the lignin polymerisation. Moreover, the peroxidases activated by ROS accumulation under Cd stress (Šípošová et al. 2021) could have increased lignin polymerisation in the Cd treatment and elevated its content in the cell walls (Tobimatsu and Schuetz 2019).

The hydrolysable part of the PF III was mainly composed of mixed glucans, GAX, and small amount of Gal. Similarly, Carpita (1983) and Suzuki et al. (2000) reported the presence of Gal bound on Ara on the side chains of GAX in the cell wall of maize. However, no attention has been paid to the non-cellulosic polysaccharides that occur only in the cell walls of type II and are firmly attached to cellulose microfibrils.

### The effects of IBA and Cd on the concentration of Cd in the polysaccharide fractions

The high fixation of Cd in the PF II can be explained by its composition (Fig. 4). PF II contained a mixture of different hemicelluloses, such as GAX, mixed-linkage glucans, xyloglucan, arabinogalactan, and a small amount of alkali-soluble pectin. The structure of pectin and GAX is composed of galacturonic and glucuronic acids that contain carboxyl groups (Krzesłowska 2011). De-esterification and de-acetylation of carboxyl groups in the GAX and pectin can create a negative charge that increases Cd binding capacity. It is known that auxin stimulates the de-methylesterification of pectin (Braybrook and Peaucelle 2013); however, its effect on the de-methylesterification of GAX is not known. The Cd could also bind to hydroxyl groups of the cell wall polysaccharides, where the ring and the bridging oxygen atoms of the monosaccharide units create negative charge (Kohn 1987).

The fixation of Cd in the PF III could be explained by the formation of complexes with oxygen atoms on the glycosidic bond and hydroxyl groups on cellulose (Moreira et al. 2013). Similar to our results, the Cd^2+^ was detected in the cellulose in *Glycine max* (Wang et al. 2018), *Brassica napus* (Wu et al. 2020), and *Arabidopsis thaliana* (Xiao et al. 2020) cell walls. In the present study, exogenous application IBA probably only indirectly influenced the Cd-fixation by cellulose, lignin, and phenols.

Both lignin (PF III) and phenols (PF I, PF II) contain carboxyl and aldehyde groups, as well as the conjugative system of the double bond of the aromatic cycle, which can bind Cd^2+^ (Vasconcelos et al. 1999; Guo et al. 2008). Application of IBA could have enhanced the synthesis of both lignin and phenols, which created more sites for Cd-binding, or regulated cell wall pH, which is a factor that influences Cd fixation (Guo et al. 2008).

### The possible mechanisms of the exogenous IBA effects on the cell wall architecture

**Fig. 5:**
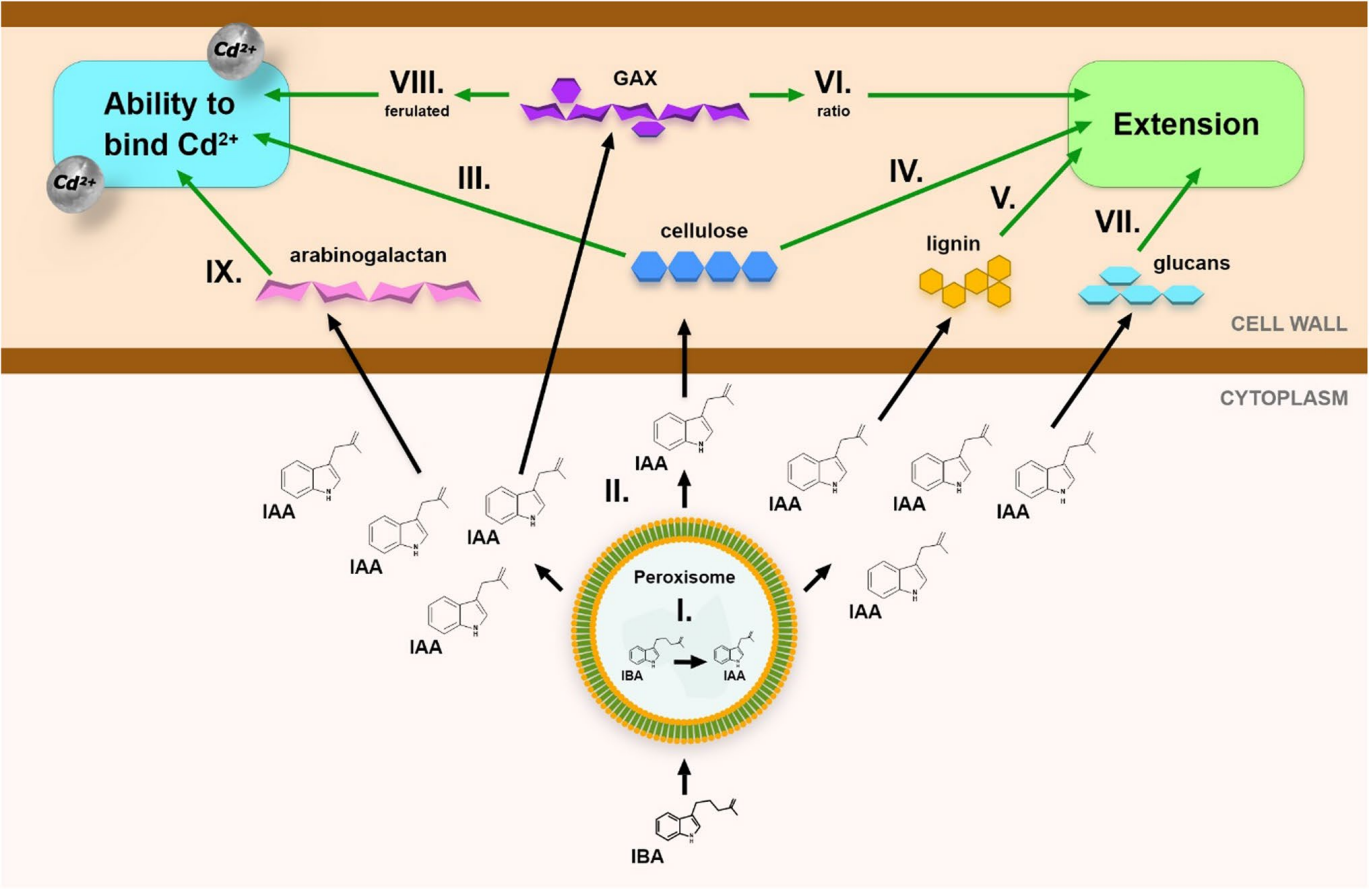
The proposed mechanisms of IBA action in the maize (*Zea mays* L.) root cell walls in the presence of Cd. (I) IBA is converted to IAA in the peroxisomes; (II) increased levels of IAA stimulated cascade of reactions in the cell wall; (III) the increased content of cellulose stimulated the fixation of Cd^2+^; (IV) a higher content of cellulose, as well as (V) the lower content of lignin, and (VI) modified ratio of GAX increased the cell wall extension; (VII) the modified content of glucans improved the cell growth and cell wall extension; (VIII) ferulated GAX increased the cell wall ability to bind Cd^2+^; (IX) the presence of arabinogalactan indicates their roles as signalling molecules in the defence mechanisms against the stress

In the light of our results, we proposed a possible mechanism of IBA action in the root cell walls exposed to Cd (Fig. 5): (I) it is suggested that IBA is converted to IAA by β-oxidation in the peroxisomes (Frick and Strader 2018); (II) the increased levels of IAA stimulated cascade of reactions in the cell wall; (III) the IAA-induced increase in the cellulose content could stimulated the fixation of Cd^2+^ within the cell wall (via hydroxyl groups) (Moreira et al. 2013). The elevated levels of IAA increased the cell wall extension and cell growth through (IV) a higher content of cellulose, (V) a lower content of lignin, and (VI) modified ratio of GAX through the increased arabinose substitution on the main chain, which keeps the cellulose microfibrils apart and allows the cell wall to extend (Shrestha et al. 2019). The improvement of cell growth could be caused by (VII) the modified content of glucans that cause irregularities during the plant growth and development; and might affect extension. Furthermore, (VIII) the cross-linking of GAX with phenols results in feruloylation of GAX (Oliveira et al. 2020), which could have increased the cell wall ability to bind Cd^2+^. IAA stimulated the content of IX) arabinogalactans in the cell walls, which indicates their roles as signalling molecules in the defence mechanisms against the stress (Kim et al. 2018). The proposed scheme has elucidated the possible mechanisms of the exogenously applied IBA and its effects on the changes in the binding of Cd^2+^ within the cell wall, and on the stimulation of growth that resulted in the amelioration of Cd stress.

## Conclusion

Our study confirmed the toxic effects of Cd on the maize (*Zea mays* L.) root anatomy and cell wall architecture. Moreover, our results show that the exogenous application of IBA, in the concentration 10^−9^ M probably alleviated the stress damage via increased levels of the endogenous IAA when compared to the Cd treatment. The positive effect of IBA application was visible in the improved plant growth, the delayed development of apoplastic barriers, and the lower content of lignin in the root cell walls. The application of IBA influenced the cell wall composition of plants exposed to Cd, increased the fixation of Cd^2+^ in the cell wall, as well as increased the retention of Cd^2+^ by hemicelluloses. The study proved the usability of exogenous application auxins as biostimulants on plants grown in the contaminated environment because the enhancement of Cd^2+^ retention in cell walls of roots could decrease the transport of Cd^2+^ to the shoots and kernels decreasing the risk of a high concentration of Cd in human food.

## Supplementary Information

ESM 1 (PDF 413 kb)

## Data Availability

Data is available on request from the authors.

## Abbreviations

Ara: Arabinose
CBs: Casparian bands
Cd: Cadmium
EM: Early metaxylem
Fuc: Fucose
Gal: Galactose
Glc: Glucose
IAA: Indole-3-acetic acid
IBA: Indole-3-butyric acid
LM: Late metaxylem
Man: Mannose
PF: Polysaccharide fraction
PME: Pectin methylesterase
PX: Protoxylem
Rha: Rhamnose
ROS: Reactive oxygen species
SL: Suberin lamellae
TC: Total content of saccharides
UA: Uronic acids
Xyl: Xylose

## Declarations

### Statement of the environmental implication

Environmental pollution by toxic compounds including heavy metals is a severe problem in many parts of the world, mainly due to excessive anthropogenic activities. Cd is a cumulative toxin that disrupts plant growth and reproduction and poses a serious health risk for animals and people. The application of natural substances, such as auxins, can be a beneficial and environmental-friendly option how to alleviate the toxic Cd effects on plants, improve their agricultural production and limit Cd entry to the food chain by the restriction of Cd movement to the consumed plant biomass.
