# Supplementary material for: The changes in the maize root cell walls after exogenous application of auxin in the presence of cadmium
Source: Environ Sci Pollut Res Int. 2023 Jul 7;30(37):87102–17. doi: 10.1007/s11356-023-28029-3 (PMC10406670; doi:10.1007/s11356-023-28029-3)

Supplementary 1. The effects of IBA on the root morphology of plants treated with Cd. Scale bar is 5 cm.

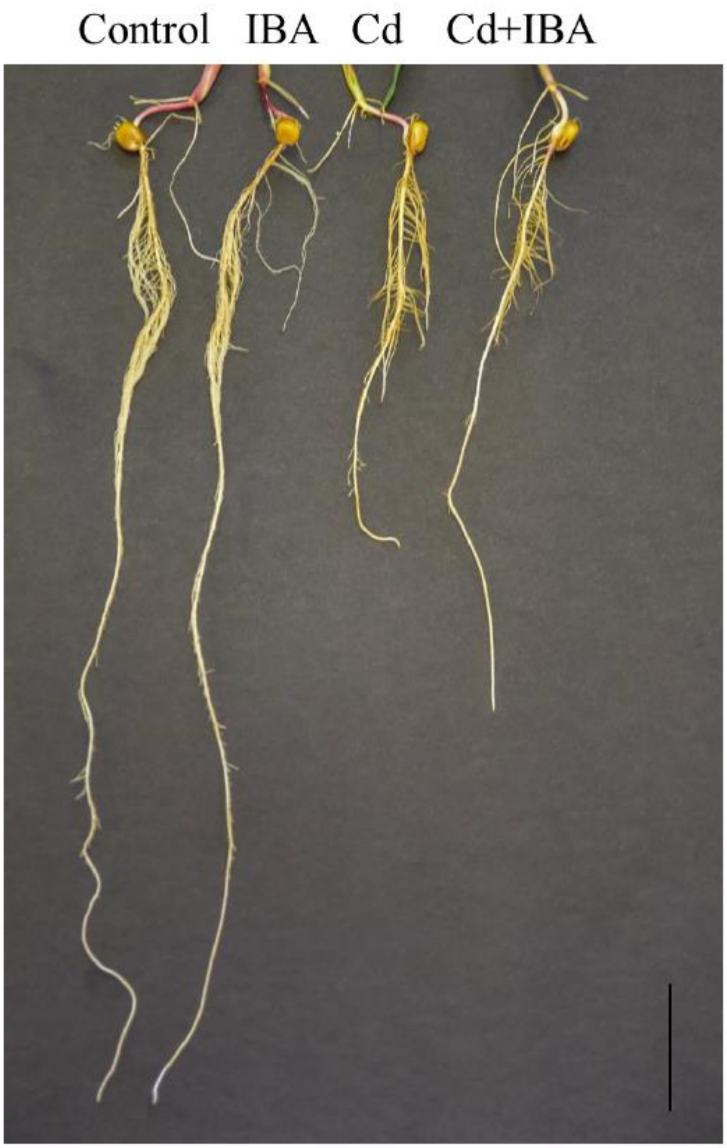

Supplementary 2. The development of the apoplastic barriers (Casparian bands (CB) and suberin lamellae (SL) in endodermis) in the maize primary root in the four treatments: Control (A), IBA (B), Cd (C), Cd+IBA (D). Cross-sections of the root were made at 50% distances from the primary root apex. Scale bar – 50 μm.

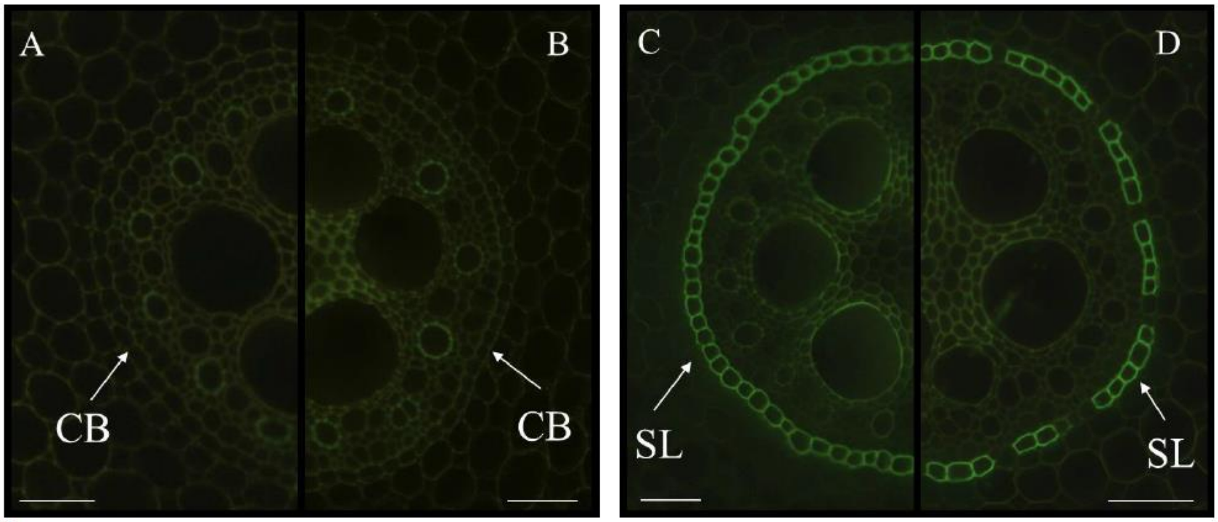

Supplementary 3. The lignification of the protoxylem (PX), early metaxylem (EX) and late metaxylem (LX) in the four treatments: Control (A), IBA (B), Cd (C), Cd+IBA (D). Cross-sections of the root were made at 40% distances from the primary root apex. Scale bar – 50 μm.

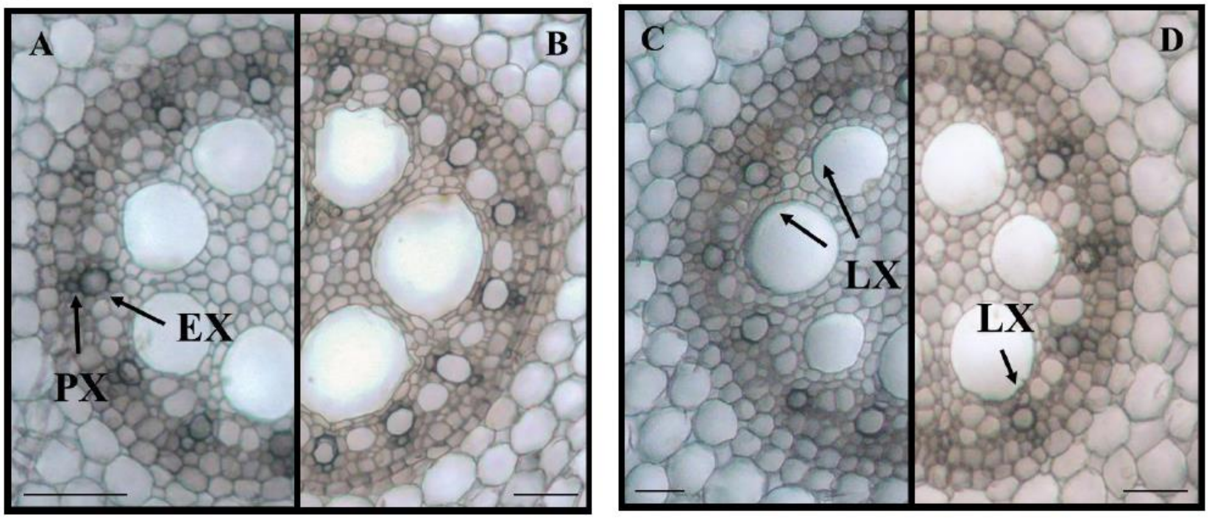

Supplement: Supplementary file 1 — (PDF 413 kb) [file 11356_2023_28029_MOESM1_ESM.pdf]
